# Potential Feasibility and Perspectives of Fecal Microbiota Transplantation for Restoring Gut Function in Giant Pandas

**DOI:** 10.3390/ani16203254

**Published:** 2026-10-16

**Authors:** Linxuan Fu, Yanzi Xue, Yanghan Wang, Shuoshuo Zhao, Qianwei Wang, Huilian Che

**Affiliations:** 1 College of Food Science and Nutritional Engineering, China Agricultural University, Beijing 100083, China; linxuan055@163.com (L.F.); xueyanzi0625@cau.edu.cn (Y.X.); 18382112879@163.com (Y.W.); zss13231986725@163.com (S.Z.); wqw202106@163.com (Q.W.); 2 China Agricultural University-Sichuan Advanced Agricultural & Industrial Institute, Chengdu 611430, China

**Keywords:** fecal microbiota transplantation (FMT), giant panda, gut microbiota, conservation medicine, microbiome engineering

## Abstract

Although the giant panda (*Ailuropoda melanoleuca*) relies almost exclusively on bamboo, its carnivore-like gastrointestinal tract has limited capacity for fiber digestion. The gut microbiota contributes to bamboo-derived nutrient utilization, although the extent of this contribution remains limited. Captivity-associated dietary and environmental changes frequently disrupt this microbial ecosystem, resulting in impaired digestive function and reduced physiological resilience. Conventional interventions, including antibiotics and single-strain probiotics, are generally insufficient to restore the complexity and stability of the gut microbiota. Fecal microbiota transplantation (FMT), which reconstructs the gut microbial ecosystem by transferring an intact microbial community, has demonstrated therapeutic potential in humans and various animal models. However, its feasibility in giant pandas remains unclear because their short intestinal tract, absence of fermentation chambers, rapid gastrointestinal transit, and host-mediated ecological filtering substantially limit donor microbiota engraftment. This review evaluates the biological rationale and technical feasibility of oral FMT in giant pandas by integrating evidence from gastrointestinal physiology, microbial ecology, and conservation medicine. This review identifies the principal barriers to microbial engraftment. It synthesizes a giant panda-specific framework from the available literature, encompassing function-oriented donor selection, optimized microbial delivery strategies, and multidimensional efficacy assessment. Collectively, this synthesis provides a theoretical foundation for future FMT research in giant pandas and offers a conceptual reference for microbiota-based interventions in the conservation of endangered wildlife.

## Simple Summary

Giant pandas feed almost exclusively on bamboo, but their digestive tract is more similar to that of carnivores. Gut microbes help pandas obtain nutrients from bamboo, yet captivity, diet changes, and disease can disrupt this microbial ecosystem, leading to poor digestion and reduced health. Fecal microbiota transplantation (FMT) transfers gut microbes from healthy donors to recipients and has shown therapeutic potential in humans and other animals. However, whether FMT can work in giant pandas remains unclear because their short intestine, rapid gastrointestinal transit, gastric acid, bile salts, and mucosal immune barriers may prevent donor microbes from establishing. This review examines the biological and potential feasibility of oral FMT in giant pandas. It identifies the main barriers to microbial engraftment and proposes a giant panda-specific framework involving function-oriented donor selection, optimized oral delivery, and multidimensional efficacy and safety assessment. These perspectives aim to guide future FMT research for giant panda conservation.

## 1. Introduction

The giant panda (*Ailuropoda melanoleuca*), an iconic species endemic to Sichuan, China, occupies a pivotal position in global biodiversity conservation. Despite substantial conservation achievements, it is currently classified as Vulnerable on the IUCN Red List [1]. Although taxonomically classified within the order *Carnivora*, fossil dental evidence from ancestral pandas indicates that the species had evolved into a bamboo-consuming omnivore approximately 3 million years ago [2]. Subsequent climate-driven habitat shifts and dietary adaptation ultimately resulted in its present-day specialization on an almost exclusively bamboo-based diet [3]. Due to its relatively short intestinal tract and the absence of a cecum, the giant panda exhibits a limited capacity to digest bamboo. Consequently, the gut microbiota contributes to compensating for these physiological limitations, although the extent of this contribution remains limited.

The gut microbiota profoundly influences the physiology, nutrition, metabolism, and growth of giant pandas and is closely associated with the development of several life-threatening diseases in this species [4,5]. Compared with healthy individuals, giant pandas suffering from enteritis exhibit significantly reduced gut microbial diversity together with a marked enrichment of opportunistic pathogens, including members of the genus *Escherichia* [6]. Gastrointestinal tract disease, especially associated with dysbiosis of gut microbiota, has been reported as a leading cause of death in giant pandas [7]. Captive giant pandas harbor significantly higher abundances and prevalence of antibiotic resistance genes (ARGs) in their gut microbiota than wild individuals do, raising concerns about the horizontal dissemination of antimicrobial resistance [8]. Although conventional antibiotic therapy can transiently suppress pathogenic microorganisms, it is generally insufficient to restore a stable gut microbial ecosystem. It may even exacerbate the loss of microbial diversity and function because of its broad-spectrum antimicrobial activity [8]. Therefore, the development of effective strategies for restoring gut microbial homeostasis has become a critical priority in giant panda conservation medicine.

Fecal microbiota transplantation (FMT) is a microbiota-based therapeutic approach that restores gastrointestinal microbial homeostasis by transferring gut microbiota from healthy donors to diseased recipients, thereby treating specific disorders and improving host health [9]. By transferring an intact microbial community, FMT has demonstrated superior efficacy in improving clinical response and remission rates in ulcerative colitis compared with other microbiome-targeted therapies in network meta-analyses [10]. In wildlife conservation medicine, however, research on and application of FMT remain in their infancy. Current veterinary applications of FMT are largely confined to companion and livestock animals. The application of FMT in the management of chronic enteropathies in companion animals, particularly dogs, demonstrated a potential to improve growth performance and intestinal health in livestock species such as pigs and cattle [9].

However, existing studies have primarily focused on characterizing gut microbial composition and the engraftment efficiency; functional performance and safety of FMT under the unique anatomical and physiological conditions of these hosts have not been investigated [10]. The lack of standardized donor selection criteria, optimized delivery strategies, and robust methods for evaluating microbial engraftment has become a major obstacle to the translational application of FMT in giant panda conservation [9].

For captive wildlife such as giant pandas, the choice of FMT delivery route is a critical practical consideration. Although distal gastrointestinal delivery, including colonoscopic infusion or rectal enema, is commonly used in companion animal practice, this route typically requires sedation or anesthesia, which poses additional health risks and handling stress in endangered species. In contrast, oral administration offers distinct advantages for giant pandas, including minimal invasiveness, reduced animal stress, elimination of anesthesia-associated risks, and suitability for repeated or long-term interventions without frequent capture or restraint [11,12]. These practical benefits make oral FMT a particularly attractive and feasible strategy for restoring the gut microbiota in captive giant pandas [9,13]. Accordingly, the present review focuses on the oral delivery route and evaluates the biological and technical feasibility of FMT in giant pandas from this perspective.

This review examines the feasibility of FMT in giant pandas from two complementary perspectives: host anatomical and physiological constraints and gut microbiota functional requirements. Specifically, it evaluates how the unique gastrointestinal characteristics of giant pandas influence the survival and engraftment of transplanted microbiota, establishes the biological rationale for FMT application, and proposes a giant panda-specific framework encompassing function-oriented donor selection, optimized microbial delivery strategies, and multidimensional efficacy assessment, thereby providing a theoretical basis for the application of FMT in giant panda conservation medicine.

## 2. Materials and Methods

### 2.1. Review Design and Search Strategy

This narrative review was conducted to evaluate the biological rationale and technical feasibility of oral fecal microbiota transplantation (FMT) for restoring gut function in giant pandas. We searched PubMed, Web of Science Core Collection, Scopus, CNKI, and Wanfang Data from database inception to 18 September 2026. The search was not restricted by publication year, but only English- and Chinese-language records were screened. The search strategy combined three core concept blocks: (i) host: “giant panda” OR “Ailuropoda melanoleuca”; (ii) microbiota: “gut microbiota” OR “gut microbiome” OR “intestinal microbiota” OR “intestinal microbiome”; and (iii) intervention: “fecal microbiota transplantation” OR “faecal microbiota transplantation” OR FMT OR “washed microbiota transplantation” OR “oral FMT” OR “capsule FMT”. Additional targeted searches were performed for gastrointestinal anatomy and transit, gastric acid, bile salts, mucosal immunity, microbiota–gut–brain axis, microbial ecology, donor selection, strain engraftment, encapsulation, biosafety, conservation medicine, and biomarkers.

### 2.2. Eligibility Criteria

Records were considered eligible if they were peer-reviewed original studies, reviews, systematic reviews or meta-analyses, guidelines, consensus statements, or relevant animal studies published in English or Chinese and addressing at least one of the following topics: giant panda gastrointestinal physiology; giant panda gut microbiota; FMT mechanisms, delivery, donor selection, engraftment, efficacy, or safety; microbiota-based interventions in wildlife or conservation medicine; or biomarkers for microbial and metabolic restoration. Records were excluded if they were conference abstracts without full text, duplicate publications, non-peer-reviewed commercial material, irrelevant to the review question, or not available in full text.

### 2.3. Screening and Selection Process

All retrieved records were exported to EndNote, and duplicates were removed. Two authors independently screened titles and abstracts according to the eligibility criteria. Full texts of potentially eligible records were then retrieved and assessed independently. Disagreements were resolved through discussion, and a third author was consulted when consensus could not be reached. A total of 64 records were finally included in this review.

### 2.4. Data Extraction and Synthesis

Data were extracted from each included record, including author, year, host species, study design, sample size, donor source, delivery route, dose, recipient pretreatment, microbial outcomes, host outcomes, and safety data. Because direct FMT studies in giant pandas are lacking, evidence from humans, laboratory animals, companion animals, livestock, and other wildlife was extracted and interpreted according to its relevance to giant panda physiology and conservation medicine. Findings were synthesized thematically into the following domains: gastrointestinal barriers to FMT, biological rationale for FMT, function-oriented donor selection, oral delivery and biosafety, and multidimensional efficacy assessment. This narrative review did not perform quantitative meta-analysis; therefore, no formal risk-of-bias scoring was applied. Evidence derived from heterologous hosts or germ-free mouse models was clearly distinguished from direct evidence in giant pandas and treated as hypothesis-generating rather than confirmatory.

### 2.5. Limitations of the Review

This review has several limitations. First, direct experimental evidence on FMT in giant pandas is extremely limited, and most conclusions are extrapolated from human, murine, or other animal studies. Second, although a structured search strategy was used, this is a narrative review rather than a systematic review, and selection bias cannot be completely excluded. Third, some relevant studies may have been published in languages other than English or Chinese or may not have been indexed in the searched databases. Finally, the proposed framework should be validated through future ex vivo, cross-species, and homologous FMT studies in giant pandas.

## 3. Impact of the Structural and Functional Characteristics of the Giant Panda Gastrointestinal Tract on FMT

### 3.1. Constraints Imposed by a Short Gastrointestinal Tract and Rapid Gastrointestinal Transit

The gastrointestinal tract of the giant panda retains the characteristic anatomical features of carnivores, with a small intestine measuring only approximately three to six times its body length [6] and the absence of a cecum [14], resulting in a limited capacity to digest bamboo. These anatomical characteristics present substantial challenges to the implementation of FMT. The residence time of donor microbiota within the recipient colon is markedly limited, restricting the time available for mucosal adhesion and ecological niche establishment, both of which are essential for successful microbial engraftment [15]. This limitation is particularly critical for strict anaerobes, which are highly oxygen-sensitive and exhibit slow growth rates, as their engraftment efficiency is strongly influenced by gastrointestinal transit time [16]. A rapid intestinal transit environment preferentially favors fast-growing microorganisms with limited colonization capacity. In contrast, microbial taxa responsible for intestinal repair are generally characterized by slower growth and stronger colonization ability, leading to competition for ecological niches between these two microbial groups [16]. Therefore, FMT capsules should be optimized through formulation modifications and appropriate dosing regimens to prolong the residence time of functional microbiota at the target intestinal niche, thereby enhancing microbial engraftment and therapeutic efficacy. A summary of these gastrointestinal characteristics is presented in Table 1.

### 3.2. Impact of the Gastric and Proximal Intestinal Environment on FMT

Orally administered FMT formulations must sequentially traverse the stomach and small intestine, where the physiological environments of these two compartments impose multiple barriers to microbial viability. The highly acidic gastric environment (with a highly acidic gastric environment in giant pandas, comparable to that of omnivorous mammals) can induce bacterial protein denaturation and DNA damage [6]. In addition, bile salts in the small intestine exert antimicrobial effects by disrupting the phospholipid bilayer of bacterial cell membranes and inducing oxidative stress [6]. These combined stresses are particularly detrimental to strict anaerobes, which generally lack antioxidant defense systems, such as superoxide dismutase and catalase, and possess cell envelope structures that are highly susceptible to bile salt-mediated damage, thereby markedly reducing their survival during passage through the stomach and small intestine [18].

Nevertheless, long-term adaptation to a bamboo-based diet may have resulted in certain physiological modifications of the giant panda gastrointestinal tract [19]. However, even with moderately reduced gastric acidity, orally administered FMT cannot completely circumvent the combined antimicrobial effects of gastric acid and bile salts [20]. Unprotected donor microbiota may undergo substantial loss of viability following gastric exposure and are subsequently subjected to further bile salt-mediated inactivation in the small intestine [21], resulting in a markedly reduced number of viable microorganisms reaching the colon relative to the initial administered dose [20].

### 3.3. Impact of the Intestinal Mucosal Immune Barrier on FMT

Even if donor microbiota successfully survive passage through the upper gastrointestinal tract and become established in the colon, their acceptance by the host mucosal immune system (MIS) remains a critical determinant of the long-term efficacy and safety of FMT [22]. Widely distributed throughout the gastrointestinal mucosa and submucosa, the MIS serves as the primary site of localized immune surveillance and regulation [22]. It maintains immune tolerance toward commensal microorganisms and dietary antigens while mounting effective immune responses against invading pathogens [22]. Theoretically, if the transplanted microbiota is recognized by the MIS as foreign antigens, inappropriate immune activation may occur, resulting in persistent low-grade intestinal inflammation.

Clinical experience with FMT in humans provides empirical evidence supporting this potential immunological risk. In a study evaluating the safety of FMT in 101 patients with active ulcerative colitis who underwent multisession FMT, the overall incidence of adverse events was 13.6%, with the most common manifestations including fever, abdominal pain, bloating, and increased stool frequency [23]. In addition, regional microbiota mismatch has been shown to induce immune dysregulation. For example, transplantation of strict anaerobes originating from the colon into the small intestine alters the local immune microenvironment and activates immune-related pathways, including Toll-like receptor (TLR) signaling and cytokine–cytokine receptor interactions, with these alterations persisting for at least three months [24].

**Figure 1:**
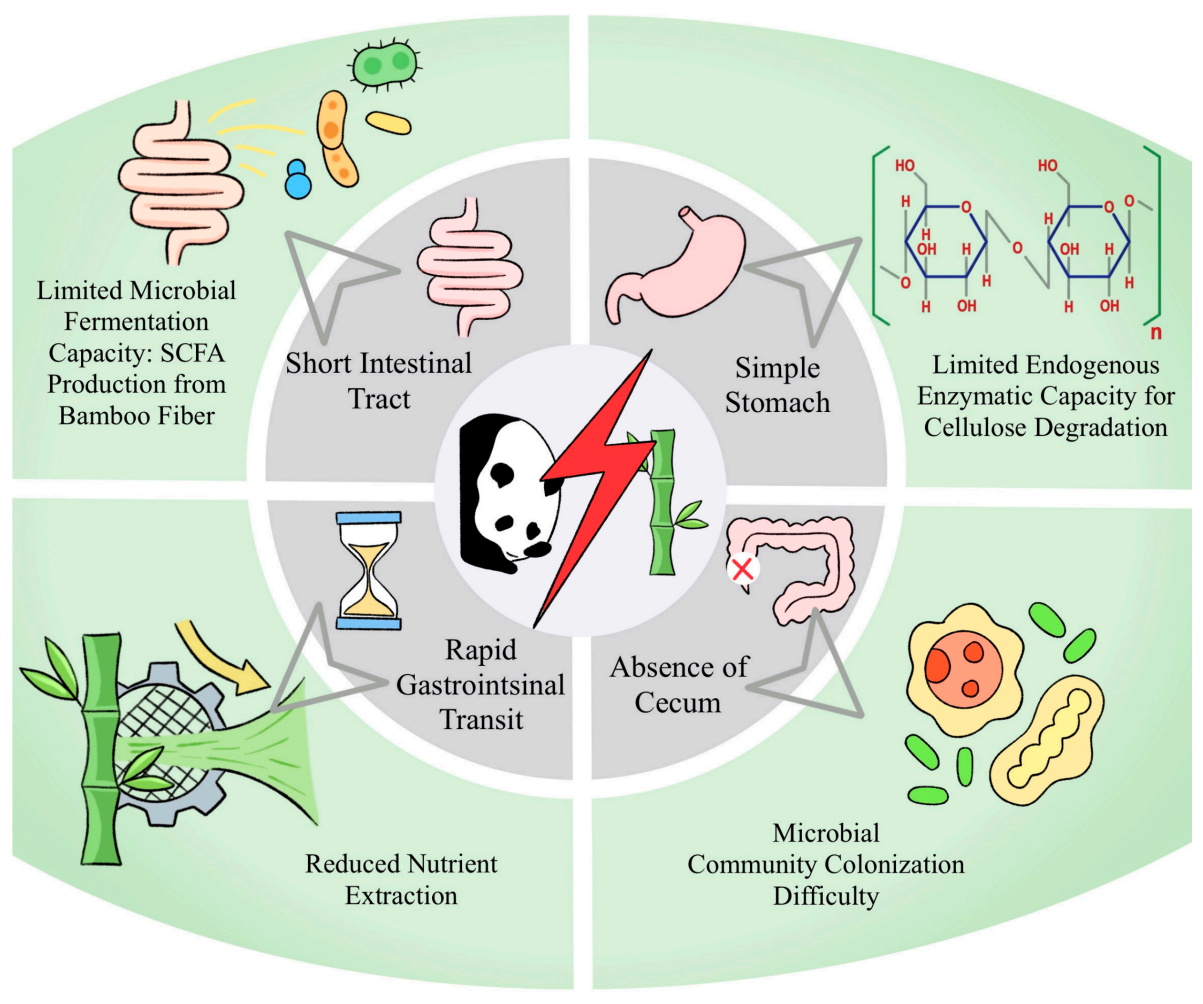
Impact of the Structural and Functional Characteristics of the Giant Panda Gastrointestinal Tract on FMT.

However, no studies have systematically evaluated the risk of inappropriate immune activation, host selection, or ecological incompatibility following FMT in giant pandas. Given that the giant panda possesses a carnivore-like gastrointestinal tract yet consumes a highly fibrous bamboo diet, its mucosal immune system may have evolved unique regulatory features that remain poorly characterized [6,25]. Figure 1 summarizes the key gastrointestinal barriers to FMT engraftment discussed in this section.

## 4. Biological Rationale for the Application of Fecal Microbiota Transplantation in Giant Pandas

### 4.1. FMT Replenishes Key Functional Microbial Taxa

Giant pandas lack the endogenous enzymatic machinery required for cellulose degradation. Nevertheless, the gut microbiota contributes to bamboo-derived nutrient utilization, although the extent of this contribution remains limited, and the giant panda should not be considered a typical highly fermentative herbivore [26]. Cellulolytic bacteria, including members of the genera *Clostridium* and *Bacillus*, constitute key functional taxa involved in bamboo fiber degradation within the giant panda gut microbiota [26]. Under captive conditions or in individuals with gastrointestinal disorders, however, the abundance and diversity of these functionally important microbial taxa may decline [7,8]. High-throughput sequencing studies have demonstrated that healthy giant pandas harbor significantly greater bacterial diversity than diseased individuals [7], with gut fungal community composition also varying with host physiological status [27], indicating a close association between host health status and gut microbial community composition.

A major advantage of FMT is that it not only replenishes depleted microbial taxa but also restores the entire gut microbial ecosystem while preserving interspecies symbiotic interactions and functional redundancy within the microbial community [9]. Moreover, the therapeutic effects of FMT may be long-lasting [28]. In a study involving patients with melanoma, fecal microbial α-diversity remained consistently higher after FMT than before transplantation, and this sustained increase was positively associated with treatment responsiveness [29,30].

### 4.2. FMT Modulates the Microbiota–Gut–Brain Axis

The microbiota–gut–brain axis (MGBA) is a bidirectional communication network that connects the central nervous system, the gastrointestinal tract, and the gut microbiota through neural, immune, endocrine, and metabolic pathways, thereby coordinating their interactions to maintain host homeostasis [31]. Bidirectional communication between the brain and the gut has been implicated in the regulation of cognition, social behavior, fear-related responses, and stress adaptation [31]. Captive giant pandas frequently exhibit low-energy behavioral patterns, including reduced locomotor activity and prolonged sleep duration, which may be influenced by the MGBA. However, this hypothesis requires validation through direct experimental studies in giant pandas.

### 4.3. Restoration of Gut Microbial Network Resilience by FMT

FMT has the potential to reconstruct the gut microbial network of giant pandas by introducing new microbial taxa, thereby enhancing community resilience [32]. Ecological theory suggests that interspecific competition generates negative feedback loops that prevent the excessive proliferation of individual taxa, thereby promoting the overall stability of the microbial ecosystem [32]. By introducing new microbial taxa, FMT may increase competitive interactions within the microbial community, reinforce these negative feedback mechanisms, and facilitate the maintenance of microbial homeostasis. The reconstructed microbial network is expected to exhibit greater functional redundancy, enabling it to better withstand environmental perturbations associated with captivity, such as dietary alterations and antibiotic exposure [33]. In contrast, simplified microbial communities are more susceptible to dysbiosis when exposed to the same disturbances, thereby increasing the risk of chronic disorders, including inflammatory bowel disease and metabolic dysfunction [34].

Given the inherently low diversity and potentially limited functional redundancy of the giant panda gut microbiota, the microbial ecosystem of this species is particularly vulnerable to perturbation under captive conditions [6,35]. Low taxonomic diversity does not necessarily imply low functional redundancy; direct evidence for the giant panda gut ecosystem remains unavailable. It should be emphasized that direct experimental evidence of FMT in giant pandas as recipients remains lacking. Current evidence is mainly derived from human studies, animal models, or germ-free mouse recipients of giant panda microbiota. Ecological theory predicts that communities with lower functional redundancy are less resilient to environmental disturbances and more prone to dysbiosis [33]. FMT, by introducing a diverse array of microbial taxa and restoring competitive network interactions, may enhance the functional redundancy and ecological resilience of the recipient gut ecosystem [33]. This positions FMT as a hypothesis for future studies, in which preventive deployment prior to the onset of overt disease may serve to reinforce ecosystem stability. From a conservation medicine standpoint, this preventive ecological rationale provides a compelling basis for the proactive application of FMT in captive giant pandas, particularly when conventional therapeutic options remain limited. It should be further emphasized that evidence obtained from germ-free mice transplanted with giant panda microbiota should remain clearly distinguished from evidence obtained directly in giant pandas, and conclusions drawn from such models should be regarded as hypothesis-generating rather than confirmatory.

## 5. Key Technical Considerations and Strategy Optimization for Fecal Microbiota Transplantation in Giant Pandas

### 5.1. Functional Niche-Based Donor Selection Strategy

The unique physiological characteristics of giant pandas dictate that donor selection for FMT should not rely solely on donor source, including whether the donor originates from wild or captive populations [8] and the specific gastrointestinal site from which the microbiota is harvested [24], but instead be guided by the principle of functional niche matching, which refers to selecting donors whose metabolic capabilities complement the underutilized ecological niches in the recipient gut [36,37].

Accumulating evidence indicates that captivity profoundly alters the functional composition of the giant panda gut microbiota. Compared with wild individuals, captive giant pandas exhibit substantially reduced cellulase activity but markedly elevated amylase activity [8]. Metagenomic analyses further corroborate these findings by demonstrating a reduced abundance of cellulose- and hemicellulose-degrading pathways accompanied by an abnormal enrichment of starch metabolism pathways in captive animals [8]. Early studies based on 16S rRNA clone libraries reported that the relative abundance of the class *Clostridia* was substantially higher in wild giant pandas (approximately 73%) than in captive individuals (approximately 42%) [26]. However, due to the technical limitations of conventional sequencing approaches, these studies were largely restricted to class-level taxonomic resolution. Microbial composition alone is therefore insufficient to accurately reflect the functional attributes required for bamboo utilization, including the abundance of carbohydrate-active enzyme families involved in cellulose, hemicellulose, and lignin degradation [36] and the capacity for short-chain fatty acid production [38]. Host-mediated detoxification of bamboo-derived secondary metabolites, such as cyanide detoxification via rhodanese activity and thiocyanate excretion [39], is an important host adaptation, but it should not be treated as a donor microbial functional trait unless direct microbiome evidence is available. This highlights the need to integrate metagenomic, transcriptomic, and functional gene profiling to comprehensively evaluate donor microbiota.

Comparative metagenomic analyses and in vitro enzymatic assays have demonstrated that several carbohydrate-active enzyme (CAZy) families involved in fiber degradation, including GH5, GH9, GH10, GH26, and GH28, are considerably less abundant in giant pandas than in typical herbivores [36]. Correspondingly, fecal cellulase and xylanase activities are also comparatively low [36]. Community composition and functional clustering analyses further reveal that the overall functional profile of gut microbiota in the giant panda more closely resembles that of carnivorous ursids than that of typical herbivores [34,36]. Moreover, microbial functions undergo pronounced seasonal dynamics during the bamboo shoot season and the bamboo leaf season, a dynamic pattern that is considered an important metabolic compensation mechanism enabling the host to adapt to changing dietary resources [36].

Importantly, recent evidence suggests that these functionally relevant microbial traits are transferable across hosts through FMT [37,38]. In a cross-species transplantation study using germ-free mice, *Clostridium butyricum*, which is enriched in the gut microbiota of giant pandas during the bamboo shoot season, was shown to promote host growth and lipid accumulation by producing butyrate, which upregulates hepatic expression of the circadian clock gene *Per2* and subsequently enhances phospholipid biosynthesis [38].

In addition to efficient fiber degradation, an ideal donor microbiota should also possess the metabolic capacity to utilize other structural components of bamboo, such as lignin [40]. Recent multi-omics analyses together with culture-based studies have revealed an enrichment of lignin-associated metabolic pathways in the gut microbiota of wild giant pandas [40]. In particular, *Pseudomonas*-associated functional modules appear to play an important role in lignin utilization, and isolated strains have been experimentally validated to degrade exogenous lignin in vitro [40]. These findings suggest that lignin degradation and aromatic compound metabolism may represent candidate functional traits for donor evaluation, although their predictive value for FMT outcomes in giant pandas remains to be validated.

A candidate donor may ideally exhibit a high abundance of CAZy families involved in cellulose, hemicellulose, and lignin degradation, demonstrate robust fermentation of bamboo-derived substrates with high short-chain fatty acid production, and satisfy stringent biosafety requirements, including a low burden of antibiotic resistance genes and the absence of specific pathogens. Furthermore, the seasonal dynamics of microbiota from wild or semi-wild giant pandas provide a valuable reference for prioritizing function-oriented donors whose metabolic capabilities match the recipient’s current dietary status. Collectively, these candidate functional traits provide a biologically grounded framework for donor selection, but their predictive value for FMT outcomes in giant pandas remains to be empirically validated. As such, they should be applied as guiding principles rather than fixed requirements, and iterative refinement based on future experimental evidence will be essential for optimizing donor selection protocols.

### 5.2. Targeted Delivery Strategies and Biosafety Considerations for FMT in Giant Pandas

The relatively short gastrointestinal tract and rapid gastrointestinal transit of giant pandas substantially reduce the residence time of donor microbiota within the colon, making this a critical determinant of successful microbial engraftment [2,6,16]. Therefore, an effective delivery system should not only protect donor microorganisms from gastric acid and bile salts during gastrointestinal transit but also maximize their colonic residence time to facilitate mucosal adhesion and ecological niche occupation.

Although no standardized delivery protocol has been established for giant pandas, experiences from human FMT provide important technical references [9]. Current human applications mainly include colonoscopic administration, oral capsules, retention enemas, and upper gastrointestinal tube infusion [41,42,43]. Among these approaches, enteric-coated oral capsules have attracted increasing attention because of their minimally invasive nature, favorable patient acceptance, and comparable therapeutic efficacy to colonoscopic delivery in recurrent *Clostridioides* difficile infection (rCDI) [41,42,43].

In addition to the delivery route, microbial engraftment after FMT is influenced by recipient pretreatment and dosing strategies, which determine the ecological conditions for donor strain persistence and colonization [44,45]. High-resolution strain-tracking studies have shown that antibiotic pretreatment and bowel cleansing can increase the probability of donor strain engraftment [44]. However, a small, randomized trial involving patients with diarrhea-predominant irritable bowel syndrome (IBS-D) reported that short-course antibiotic pretreatment reduced long-term donor strain persistence [45]. These apparently conflicting findings indicate that no universally optimal pretreatment strategy currently exists, and its effectiveness is likely to depend on disease status, the baseline microbial community, and host physiological characteristics [44,45].

To overcome the challenges posed by rapid gastrointestinal transit, recent advances in enteric micro-encapsulation technologies offer promising solutions for FMT delivery [43,46]. Multilayer encapsulation systems based on natural polymers such as alginate, chitosan, and gelatin can protect anaerobic microorganisms from gastric acidity, enable pH-responsive colonic release, and prolong microbial residence on the intestinal mucosa through bioadhesive interactions [47,48]. In addition, micro-encapsulation techniques including spray drying, ionic gelation, and layer-by-layer self-assembly have improved the uniformity of particle size and pore structure, providing an engineering platform for controlled and sustained microbial release [49].

Washed microbiota transplantation (WMT), which employs automated processing to remove undigested food particles, fungal spores, and selected pro-inflammatory metabolites, has demonstrated clear advantages over conventionally filtered fecal suspensions by improving sample consistency and reducing the incidence of adverse events [50]. Standardized operating procedures have subsequently been established through the *Nanjing Consensus*, which provides guidance on donor screening, washed microbiota preparation with automatic facilities, storage and transport, patient preparation, delivery route selection, and safety management [51]. In parallel, international stool banks such as OpenBiome have implemented comprehensive quality management systems encompassing donor screening, sample processing, cold-chain transportation, and full-process traceability, thereby providing valuable models for standardized FMT practice [52]. Furthermore, the U.S. Food and Drug Administration (FDA) has repeatedly issued safety alerts regarding the transmission of extended-spectrum β-lactamase (ESBL)-producing organisms and carbapenem-resistant Enterobacteriaceae (CRE), prompting the implementation of stricter donor screening and antimicrobial resistance surveillance protocols [53].

### 5.3. A Multidimensional Framework for Evaluating FMT Efficacy: Integrating Metabolic Homeostasis and Host Immune Responses

**Figure 2:**
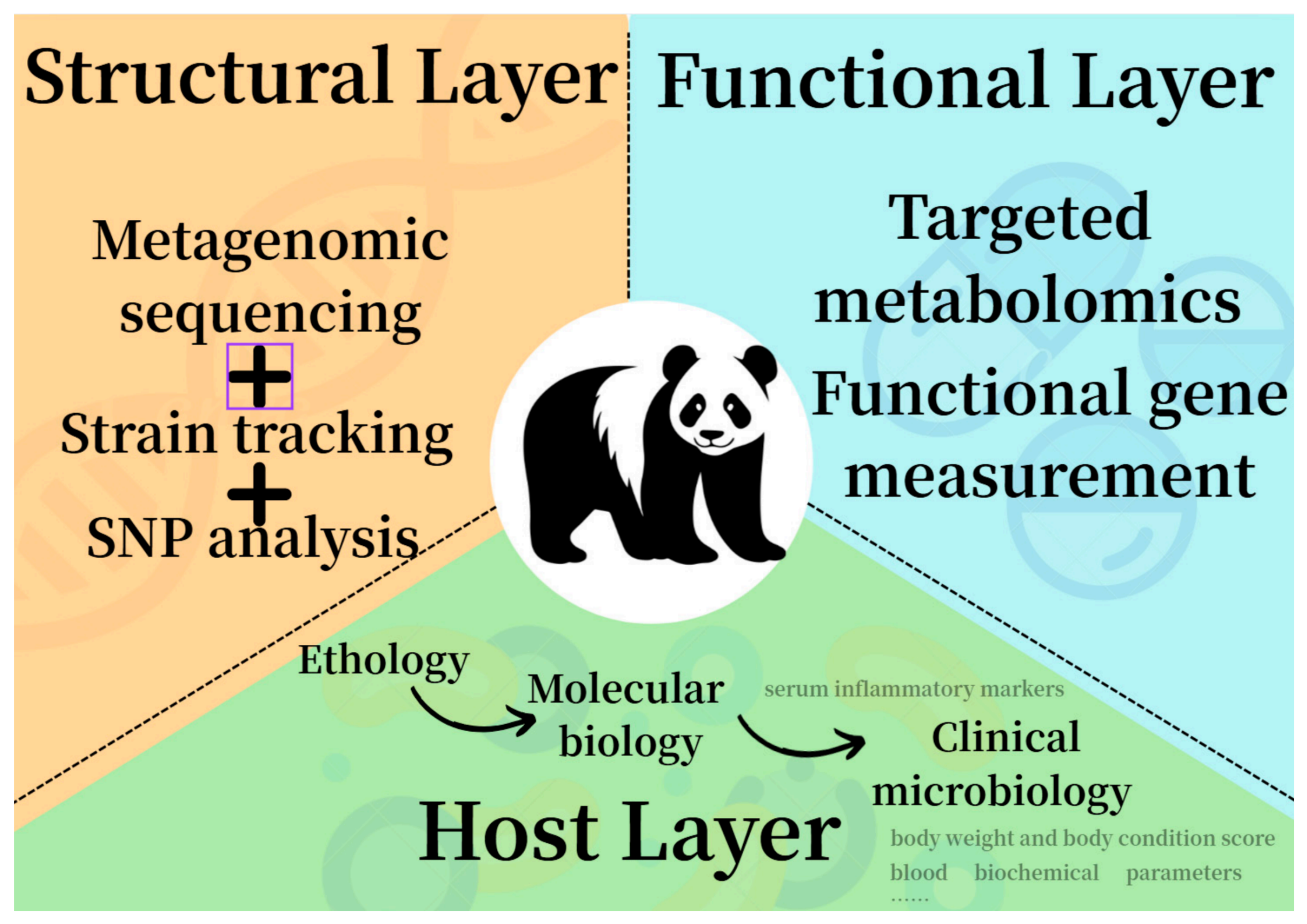
Three-dimensional framework for evaluating FMT efficacy.

Cross-species FMT studies are frequently hindered by the absence of standardized evaluation criteria, limiting comparisons across studies [54]. This challenge is particularly pronounced in giant pandas, for which no gold-standard framework exists to distinguish true ecological engraftment from the transient passage of donor microorganisms. To address this methodological gap, the present review outlines a multidimensional evaluation framework tailored to the physiological characteristics of giant pandas, integrating structural, functional, and host-response assessments that span from microbial mechanisms to host phenotypes, as illustrated in Figure 2 and detailed in the following subsections.

#### 5.3.1. Structural Assessment: From Community Composition to Strain-Level Engraftment

Owing to host-mediated ecological filtering [6,18], the engraftment efficiency of donor microbiota in giant pandas is inherently constrained. Evidence from germ-free mouse models suggests that only approximately 9% of giant panda donor genera successfully re-establish in the recipient gut, substantially lower than the 32% observed for equine donor microbiota [55]. However, these estimates should be interpreted with caution, as they were obtained from a heterologous host (mouse) rather than from conspecific giant panda recipients, and the germ-free mouse gut lacks colonization resistance from resident microbiota, which may affect the quantitative extrapolation of engraftment rates to giant pandas. Therefore, the 9% engraftment rate should be regarded as a model-specific reference rather than a direct prediction of FMT efficacy in giant pandas. Nevertheless, taxonomic profiling at the species level cannot distinguish between transient donor passage and stable ecological engraftment, because multiple strains of the same species may differ substantially in their colonization capacity and functional traits [56,57]. Therefore, higher-resolution approaches, such as strain-level tracking, are required to quantify donor strain engraftment and persistence accurately [56,58,59]. Therefore, structural evaluation should incorporate shotgun metagenomic sequencing coupled with high-resolution strain-tracking pipelines, such as StrainPhlAn, bioBakery3, or inStrain, to reconstruct strain-level phylogenetic relationships and single-nucleotide polymorphism (SNP) profiles. Such analyses enable quantitative assessment of donor strain engraftment, persistence, and replacement dynamics within pre-existing microbial niches [56,58,59].

#### 5.3.2. Functional Assessment: Metabolic Reconstruction and Restoration of Ecological Functions

Functional assessment aims to determine whether FMT restores microbial fermentation capacity and metabolic homeostasis in the recipient [60]. First, the total concentration of short-chain fatty acids (SCFAs), particularly the proportion of butyrate, reflects colonic fermentation efficiency and local energy metabolism. Quantification using isotope-labeled internal standards is recommended to improve the robustness of absolute metabolite measurements [61]. Second, multiple clinical studies have demonstrated that recovery of secondary bile acid production following FMT is accompanied by restoration of bile salt hydrolase (BSH) activity and expression of the bai operon, with these metabolic improvements being strongly associated with favorable clinical outcomes [62]. Given the apparent conservation of this metabolic response across host species, the recovery of secondary bile acids, particularly deoxycholic acid (DCA) and lithocholic acid (LCA), has been proposed as a candidate biomarker of functional microbiota restoration, as demonstrated by studies showing that FMT consistently restores normal fecal secondary bile acid composition in patients with recurrent *Clostridioides difficile* infection [37,63,64]. However, it is important to acknowledge that most evidence supporting this indicator originates from human CDI studies, and the giant panda differs substantially from humans in terms of dietary composition, gastrointestinal anatomy, and baseline bile acid metabolism. Therefore, while secondary bile acid recovery represents a promising candidate biomarker for functional restoration, it should be considered an exploratory endpoint requiring validation in giant panda-specific contexts rather than a directly transferable validated indicator.

#### 5.3.3. Host Assessment: Physiological Rhythmicity and Systemic Safety

The success of FMT should be evaluated according to its physiological benefits and safety in the host. Experimental evidence has established a causal relationship between seasonal changes in the gut microbiota and host metabolic rhythmicity, demonstrating that butyrate-enriched microbiota from the bamboo-shoot feeding season promote phospholipid biosynthesis and lipid accumulation by upregulating the expression of the peripheral circadian clock gene *Per2* [38]. Accordingly, restoration of circadian rhythmicity, including activity rhythm indices and expression profiles of core clock genes, should be incorporated as sensitive endpoints for assessing the extent of microbiota–host functional interactions [38].

In addition to these molecular and circadian markers, the framework should also incorporate direct indicators of intestinal functional recovery that are readily measurable in captive management settings. These include fecal consistency and frequency, diarrhea incidence, appetite and bamboo intake, body weight and body condition score, serum inflammatory markers, blood biochemical parameters, and systematic recording of adverse events. In giant pandas, reduced appetite and altered fecal characteristics are well-documented clinical signs of gastrointestinal distress and dysbiosis [6,7]. Body weight and body condition have been shown to respond to gut microbiota composition in cross-species transplantation models [38]. Inflammatory and immune responses following FMT may reflect host–microbiota interactions [35], and safety monitoring through adverse event recording is a standard component of FMT studies [23]. These host-level outcomes complement the structural and functional microbial assessments, providing practical, clinically relevant evidence for evaluating both the efficacy and safety of FMT in giant pandas.

Concurrently, a comprehensive biosafety surveillance system integrating microbial culture with molecular diagnostics should be established to monitor multidrug-resistant organisms, particularly ESBL-producing bacteria and CRE [53]. Detection of these organisms or the occurrence of persistent systemic adverse events, such as sustained fever, should trigger immediate batch recall and traceability investigations.

## 6. Conclusions and Perspectives

The current body of evidence indicates that the feasibility of fecal microbiota transplantation (FMT) in giant pandas is determined not simply by the transfer of donor microorganisms but by the successful establishment of microbial functions within a highly specialized gastrointestinal ecosystem. The carnivore-like digestive anatomy, rapid gastrointestinal transit, and low-diversity gut microbiota together create substantial barriers to donor microbiota engraftment while simultaneously making microbiota-mediated metabolism indispensable for bamboo utilization and host health. These characteristics distinguish giant pandas from both conventional veterinary species and human FMT recipients, highlighting the need for species-specific strategies rather than direct translation of existing clinical protocols.

Based on the available evidence, this review proposes an integrated conceptual framework for giant panda FMT that connects three essential components: function-oriented donor selection, optimized microbial delivery, and multidimensional evaluation of microbial engraftment and host responses. Rather than representing independent technical procedures, these components should be considered an integrated workflow for improving ecological compatibility between donor microbiota and the recipient intestinal environment. This framework provides a biological rationale for designing future FMT studies in giant pandas and offers a structured approach for evaluating treatment outcomes.

At present, the major obstacles to clinical translation are no longer the theoretical feasibility of FMT but the lack of direct experimental evidence in giant pandas. Based on the anatomical, physiological, and microbial barriers discussed above, we consider the following strategies to be the most feasible near-term approaches for improving FMT success in giant pandas. First, optimizing delivery systems to prolong donor microbiota residence in the colon is a feasible approach. For instance, enteric-coated capsules or micro-encapsulation technologies could mitigate the rapid gastrointestinal transit that limits engraftment [43,46,47,48]. Second, prioritizing donor selection based on functional traits rather than donor origin alone, as outlined in Section 4.1, may improve ecological niche matching between donor and recipient. Third, combining FMT with appropriate recipient preconditioning, such as short-term dietary adjustment or, where indicated, targeted antibiotic pretreatment, may reduce colonization resistance and create a more permissive gut environment for donor microbiota establishment [46,47]. Fourth, given the current absence of direct evidence in giant pandas, we advocate a step-wise translational roadmap: beginning with ex vivo fermentation assays using bamboo-derived substrates to screen donor functionality, followed by cross-species transplantation in germ-free mouse models to evaluate engraftment potential and metabolic effects [38,55], and ultimately progressing to homologous FMT trials in captive giant pandas with rigorous safety monitoring and multidimensional outcome assessment as proposed in Section 4.3. This incremental approach would allow systematic optimization of protocols while minimizing risks to this endangered species.

Addressing these questions will establish the evidence required for microbiota-based interventions in giant panda conservation and may provide a useful framework for other wildlife species with specialized diets and highly host-dependent gut microbial ecosystems.

## Figures and Tables

**Table 1:** Structural and Functional Characteristics of the Giant Panda Gastrointestinal Tract Relevant to Fecal Microbiota Transplantation.

Characteristic	Generalist Carnivores	Giant Panda	Hindgut Fermenters (e.g., Horse)
Relative intestinal length	Short	Short [6]	Long
Cecal development	Poorly developed	Absent [6]	Well developed; serves as the primary site of microbial fermentation
Gastric structure	Simple stomach with highly acidic gastric juice	Simple stomach with relatively milder gastric acidity [6]	Simple stomach in some species; multi-chambered stomach in ruminants
Mean gastrointestinal transit time	Short	Short [17]	Long
Primary digestive strategy	Enzymatic digestion	Limited post-gastric microbial fermentation [6,17]	Predominantly hindgut microbial fermentation

## Data Availability

No new data were created or analyzed in this study. Data sharing is not applicable to this article.
